# Amnion and Chorion Membranes: Potential Stem Cell Reservoir with Wide Applications in Periodontics

**DOI:** 10.1155/2015/274082

**Published:** 2015-12-06

**Authors:** Akanksha Gupta, Suresh D. Kedige, Kanu Jain

**Affiliations:** ^1^Department of Periodontics, Maharishi Markandeshwar College of Dental Sciences and Research, Mullana, Ambala, Haryana 133207, India; ^2^Department of Oral Pathology, Jaipur Dental College, Jaipur, Rajasthan 303805, India

## Abstract

The periodontal therapy usually aims at elimination of disease causing bacteria and resolution of inflammation. It involves either resective or regenerative surgery to resolve the inflammation associated defects. Over the years, several methods have been used for achievement of periodontal regeneration. One of the oldest biomaterials used for scaffolds is the fetal membrane. The amniotic membranes of developing embryo, that is, amnion (innermost lining) and chorion (a layer next to it), have the properties with significant potential uses in dentistry. This paper reviews the properties, mechanism of action, and various applications of these placental membranes in general and specifically in Periodontics.

## 1. Introduction

**Figure 1:**
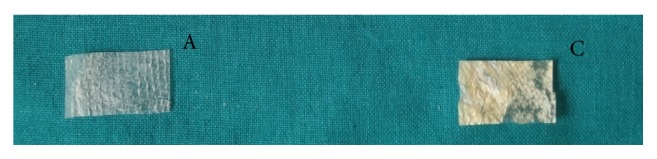
Lyophilized gamma irradiated amnion (A) and chorion (C) membranes, prepared to be used in periodontal therapy.

The ultimate goal of periodontal treatment using regenerative techniques is to restore the supporting tissues lost as sequelae of inflammatory periodontal disease [1]. Different methods have been used in the past for the achievement of this goal. These procedures include root planing, soft-tissue curettage, and flap surgeries. The latter procedures have often been used along with grafting of bone, guided-tissue regeneration, incorporation of biomaterials like derivatives and substitutes of bone, and biologic factors like enamel matrix proteins [2]. One of the new materials which has also been tried recently includes placental membranes. The placental allografts possess antibacterial and antimicrobial properties being tissues with immunoprivilege and are thus quite different from cadaveric allograft, xenograft, and alloplast barrier membranes used in periodontal therapy [3]. They reduce inflammation and provide a matrix highly rich in protein and thereby facilitate migration of cells at the area of defect [3]. Applications of amnion membrane include chemical or thermal burns, correction of corneal epithelial defects, neurotrophic corneal ulcers, leaking blebs after glaucoma surgery, reconstruction of conjunctival and ocular surfaces, ocular cicatricial pemphigoid or Stevens-Johnson syndrome, and bullous keratopathy [4]. In Periodontics, these membranes have also been used in furcation defects, intrabony defects, and gingival recession coverage (Figure 1). In this literature review, the various properties of the placental membranes are discussed in light of their potential uses in the field of Periodontics.

## 2. Historical Background

Human amniotic membranes have been used successfully for a wide range of applications for over 70 years. The use of fetal membrane in skin transplantation was first reported by Davis in 1910 [5]. Description of the use of human amniotic membrane for burned and ulcerated skin surfaces was given by Stern in 1913 [6]. They evaluated the accelerative effect of the membrane on epithelialization and the reduction in pain by its application on burned or ulcerated sites [7]. In 1940, De Röth [8] first reported use of fetal membranes in the ocular surface. He used fresh amnion and chorion as a biological dressing material for management of conjunctival defects. Kim and Tseng gave the preservation method for maximal maintenance of biologic properties of membranes which is still the best way [9]. The amniotic membrane has gained importance specifically because of various factors. Firstly, it reduces scarring and inflammation and enhances wound healing. Secondly, it serves as a scaffold for proliferation and differentiation of cells owing to its antimicrobial properties. Thirdly, its extracellular matrix and its components, such as growth factors, suggest it to be an excellent biomaterial to act as a native scaffold for tissue engineering. Lastly, it can be easily procured, processed, and transported.

## 3. Anatomy and Histology

Placental membranes have their origin from extraembryonic tissue. This tissue is composed of a fetal component (the chorionic plate) and a maternal component (the deciduas). The fetal component includes the amnion and chorion membranes which separate the fetus from the endometrium. The structure of amniotic membrane has three parts which are epithelial monolayer, a thick basement membrane, and an avascular stroma.

Epithelial monolayer consists of a single layer of cells which are arranged uniformly on the basement membrane. It is the innermost layer, lies nearest to the fetus, and is also called the amniotic epithelium. The amniotic epithelial cells have an active secretory and transport functions as suggested by their ultrastructure [10]. This epithelium is firmly fixed to a basement membrane which is in turn attached to a condensed acellular layer. This layer is composed of collagen types I, II, and V [10]. Blood vessels or nerves are absent in amniotic membrane. It derives its nutrition directly by diffusion out of the amniotic fluid.

The basement membrane is quite remarkable as it is one of the thickest membranes found in all human tissues and provides support to the fetus throughout gestation [11]. It is similar to that of conjunctiva in its distribution of collagen type IV subchains.

**Figure 2:**
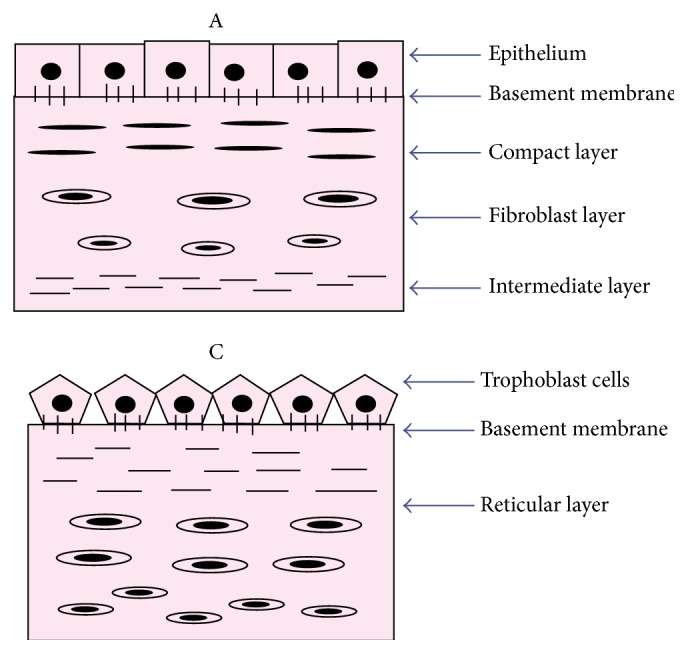
Line diagrammatic representation of histological architecture of amnion (A) and chorion (C) membranes.

The main fibrous skeleton of amniotic membrane is formed by the compact layer of stroma lying adjacent to basement membrane. Next layer that is fibroblastic layer containing mesenchymal cells is responsible for secretion of different types of collagens. Predominant types are interstitial collagens (types I and III) which form parallel bundles to maintain the mechanical integrity of the membrane. Filamentous connections between interstitial collagens and epithelial basement membrane are provided by collagens types V and VI. The last layer which is known as intermediate layer or spongy layer or zona spongiosa lies adjacent to the chorionic membrane and contains a meshwork of mostly type III collagen [12]. Its spongy appearance is the result of presence of abundant content of proteoglycans and glycoproteins. Chorion is composed of reticular layer, basement membrane, and trophoblasts (Figure 2) (Table 1).

## 4. Principle of Therapeutic Tissue

The preparation of placental extract was described by Russian ophthalmologist, Professor VP Filatov. Though its use was popular in Europe and parts of Asia, primarily China, Korea, and Japan, for over a century, there was no documentation on its therapeutic efficacy prior to Filatov's research. An increased surge in research on human placental extract took place after his description. Filatov started research on grafting human corneas by using the principle of transplantation of preserved material. He observed that animal or vegetable tissues undergo a biochemical readjustment after their isolation from the organism and subjection to environmental factors that inhibit their vital processes. As a result, the tissues start developing substances to stimulate their vital processes. These substances were termed as biogenic stimulators by Filatov [13].

After many experiments of Filatov, he was convinced that any human or animal tissue which may not necessarily correspond histologically to pathological tissue could be used to obtain curative effect. He extended this concept to general medicine which later gave birth to principle of therapeutic tissue. He confirmed the process to be just as valid for other human tissues [14].

## 5. Properties of Membranes (Table 2)

### 5.1. Biomechanical Properties

Thickness of normal amniotic membrane lies between 0.02 and 0.5 millimetres which includes around 6–8 layers of cells. An average surface area of this membrane is about 1600 square centimetres [15]. An important property of amniotic membrane is its resistance to various proteolytic factors owing to the presence of interstitial collagens [16]. Elastin present in amnion is responsible for providing elasticity. It has multiple metabolic functions such as its role in water and soluble material transportation and production of bioactive peptides, growth factors, and cytokines [17].

### 5.2. Promotion of Epithelialization

Amniotic membrane facilitates migration of epithelial cells [18], reinforces basal cell adhesion [19], promotes epithelial differentiation [20], prevents epithelial apoptosis [21], and promotes epithelialization in healing of wounds [22]. Various growth factors produced by amniotic membrane can stimulate epithelialization [23]. It can also promote expansion and maintenance of epithelial progenitor cells in vivo [24] and can produce endothelin-1 and parathyroid hormone related protein. Brain natriuretic peptide and corticotrophin releasing hormone are also produced by membrane epithelial cells which play roles in increasing cellular proliferation and calcium metabolism [25]. Expression of mRNA for epidermal growth factor, hepatocyte growth factor receptor, and keratocyte growth factor receptor was demonstrated by Koizumi et al. in 2000 in cryopreserved amniotic membrane [23]. Its basement membrane serves as a safe and suitable bed for the growth of epithelial cells. Sufficient oxygenation for epithelial cells is provided by its good permeability in contrast to other synthetic materials. Thus, amniotic membrane is an ideal tissue which facilitates the growth of epithelial cells, helping in their migration and differentiation [26, 27].

### 5.3. Inhibition of Fibrosis

The amniotic membrane possesses antifibrosis property. Fibroblasts are naturally responsible for scar formation during wound healing and are activated by transforming growth factor *β*.

Amniotic membrane reduces the risk of fibrosis by downregulation of transforming growth factor *β* and its receptor expression by fibroblasts. Therefore, scaffold of an amniotic membrane modulates wound healing by promoting reconstruction of tissues rather than promoting formation of scar tissue [28, 29].

### 5.4. Inhibition of Inflammation and Angiogenesis

The exact mechanism of the anti-inflammatory properties of amniotic membrane is not clear. It is hypothesized that it decreases influx of inflammatory cells to the wound area and consequently reduces inflammatory mediators by serving as a barrier. It entraps T lymphocytes when it is applied as a patch in vivo [30]. Matrix metalloproteinases released by infiltrating neutrophils and macrophages are taken care of by inhibitors of matrix metalloproteinases found in the amniotic membrane. Presence of various tissue inhibitors of metalloproteinases 1, 2, 3, and 4, interleukin-10, and interleukin-1 receptor antagonists and endostatin which inhibit endothelial cell proliferation, angiogenesis, and tumor growth has also been observed in amniotic membrane [31]. The presence of proteinase inhibitors may facilitate wound healing [32]. Thrombospondin-1, secreted by the amniotic epithelium, acts an antiangiogenic factor. Two very potent proinflammatory mediators, interleukin-1*α* and interleukin-1*β*, are suppressed by matrix of stroma of amniotic membrane [33]. Shimmura et al. in 2001 reported that amniotic membrane reduces inflammation through entrapment of inflammatory cells [30]. A high molecular-weight glycosaminoglycan, hyaluronic acid, present in large quantities in amniotic membrane acts as a ligand for CD44 which is expressed on inflammatory cells. It plays an important role in adhesion of inflammatory cells including lymphocytes, to the amniotic membrane stroma [34].

Other substances expressed in the amniotic membrane are low-molecular-mass elastase inhibitors which include secretory leukocyte proteinase inhibitor and elafin [35, 36]. These inhibitors have antimicrobial actions in addition to their anti-inflammatory properties [37]. They protect related surfaces from infection, thereby acting as components of the innate immune system [37]. Both antimicrobial and anti-inflammatory properties can also be induced into amniotic membranes by their treatment with both lactoferrin and interleukin-1 receptor [38]. Lactoferrin, a globular multifunctional protein, has both antimicrobial and anti-inflammatory qualities. It serves as an antioxidant and an iron chelator in tissues [39] and suppresses the production of interleukin-6 in the amniotic fluid during amniotic infection. Interleukin-1 receptor antagonist on the contrary reduces inflammation as it is a potent inhibitor of interleukin-1 which is a mediator of inflammation [40].

### 5.5. Lack of Immunogenicity

Occurrence of acute rejection after transplantation of amniotic membranes is negated by the fact that amniotic epithelial cells do not express HLA-A, HLA-B, HLA-D, and HLA-DR antigens but express HLA-G on their surfaces [41]. Presence of interferon *γ* and other immunologic factors has also been observed in the amniotic membrane. It seems that amniotic membrane may induce immunologic reactions in the presence of viable epithelial cells. One study revealed that transplantation of fresh amniotic membrane is associated with a mild inflammatory response. This could be probably due to expression of HLA-I antigens by viable epithelial cells [42]. However, immunogenicity of cryopreserved amniotic membrane is less than that of fresh amniotic membrane as epithelial cells are lost in cryopreservation [43]. T lymphocytes in allografted limbus cells are suppressed by amniotic membrane. This implies immunosuppressive properties of amniotic membrane which can increase the chances of successful grafting [44]. As tissue grafts of placental membrane materials present a low risk of immune rejection, they are considered to be bestowed with “immune privilege” [45, 46].

### 5.6. Antimicrobial and Antiviral Properties

The risk of infection is reduced by amniotic membrane due to its antimicrobial and antiviral properties [47]. Microorganisms upon their entry into the body are eliminated by our immune system through an adaptive immune response, *β*-defensins, a major group of antimicrobial peptides and an integral part of the innate immune system, which are expressed at surfaces of mucosa by epithelial cells and leukocytes [48, 49]. Amniotic membranes also have the ability to produce *β*-defensins [35] with the predominant type present in amniotic epithelium being *β*3-defensin [40]. Kjaergaard et al. in 2001 have also shown in vitro antimicrobial effects of the amnion and chorion against certain microorganisms. Its antiviral properties are exhibited by presence of cystatin E, the analogue of cysteine proteinase inhibitor [50]. There is still further need for studies to verify these properties of the amniotic membrane [51]. Amniotic membrane may prevent infiltration and adhesion of microorganisms to wound surfaces by acting as a barrier. The hemostatic property of collagen fibers of amniotic basement membrane prevents hematoma formation in clean surgical wounds. This reduces bacterial load and risk of infection by preventing accumulation of microbes. Another mechanism of action against infection by membranes is through their adhesion to the wound surface. This attachment prevents formation of dead space and accumulation of serous discharge. Furthermore, bacterial entrapment and stimulation of migration of phagocytes also occur by fibrin filaments formed during wound healing. These filaments cause adhesion of the wound bed to amniotic membrane collagens. There is a report that bacterial proliferation is reduced even in contaminated wounds by amniotic membrane [52].

### 5.7. Cell Differentiation Property

The fetal placental tissues have the potential to transform into different cell lineages. The hematopoietic lineage is found in the chorion, allantois, and yolk sac; and the mesenchymal lineage is found in both the chorion and amnion. The cells isolated from the chorion are good sources of cells of hematopoietic and mesenchymal lineages as they possess these properties. It is considered that the amniotic membrane can maintain pluripotent stem cell potential for cell differentiation.

## 6. Applications

Amniotic membrane can be used for transplantation either as a temporary graft or as a permanent graft. It can be used alone or in conjunction with other surgical procedures when employed as permanent graft. In temporary grafting procedure, it is sutured to both healthy host tissue and site of interest at the same time as a bandage or dressing, or patch. This is so done to promote healing of host epithelial lining lying underneath. The membrane is invariably dissolved once epithelialization is complete. The removal is carried out in a period varying from 2 to 6 weeks. When used for permanent grafting, for example, in cornea or conjunctiva, it is sutured to fill in the tissue defect so that host cells proliferate into it and a sound integration with the host tissue is achieved.

## 7. Applications of Placental Membranes Based on Their Properties

1. The physical properties of amniotic membrane have proven it to be compatible with corneal surface of the eye. The eye and placental membranes have so much similarity in their immune modulatory properties that they have been referred to as “parallel universes” [53].
2. The use of human amniotic membrane as a surgical wound dressing in treatment of leg ulcers, skin loss in Stevens-Johnsons diseases, reconstruction of the pelvic floor, vaginal epithelialization, replacement of normal mucosa in Rendu-Osler-Weber diseases, and ear surgery has been described [54]. Amniotic membrane acts as a biologic dressing resulting in significant pain relief in burns owing to adhesion to the wound surface and coverage of dermal nerve endings. It also prevents wound surface drying, which accelerates wound healing, and thus can be used to manage wounds in the oral cavity like that of tongue, buccal mucosa, vestibule, palatal mucosa, and floor of the mouth [55, 56].
3. Amniotic membrane is considered as an important potential source for scaffolding material that must easily integrate with host tissue and provide an excellent environment for cell growth and differentiation. The extracellular matrix components of the basement membrane have a great role in cell adhesion during the cell seeding protocol.
4. Human amniotic membrane is also used as a potential dressing that accomplishes four major goals:
  - Haemostasis.
  - Reduction of water loss through evaporation, by acting as water barrier, and providing a moist environment for cell survival and growth.
  - Acting as barrier to microbial colonization and infection.
  - Reduction in pain.
5. Talmi et al. in 1990 have reported the use of human amnion for overlying epithelial defects after flap necrosis following surgery in the head and neck region with good results [54].
6. Florian et al. in 2013 tried amniotic membrane as an interpositional material for the reconstruction of TMJ ankylosis as it has antifibrotic properties and thus can prevent reankylosis [57].
7. Owing to its antimicrobial properties, amniotic membrane can be even used as a carrier for local delivery of the various drugs [58].

## 8. Applications in Periodontics

*(i) Preclinical Studies*

(a) Gomes et al. in 2001 studied the use of amnion grafts to line the floors of cortical bone defects and to cover the superficial surface of the defects. At 90 days, amnion tissue was in direct apposition to newly formed bone [59]. At 120 days, the amnion tissue grafts were no longer present and bone had completely filled the defects. The authors concluded that the use of amnion tissue grafts did not inhibit repair in guided bone regeneration and may have been beneficial for its antibacterial properties.
(b) Rinastiti et al. in 2006 histologically evaluated the use of amnion tissue in thirty 3-4-month-old rabbits. Amnion tissue grafts in this study were made by layering 5 sheets (5 × 5 mm) of freeze-dried, human amniotic membrane [60]. Half of the wounds were covered with amnion grafts and the other half of the wounds served as the uncovered, control group. Compared to the control group, the amnion treated wounds had fewer polymorphonuclear cells at days 1 and 3; thicker epithelium and more fibroblasts at days 5, 7, and 10; statistically significant greater new blood vessel formation at days 7 and 10; and significantly more mature and dense collagen fibers at day 10.
(c) The treatment of oral mucositis in rats with amniotic membrane was studied by Vilela-Goulart et al. in 2006 [61]. The amnion treated group demonstrated hypercellularity, including endothelial cells and fibroblasts, and intense vascularity. In addition, amnion treatment had accelerated healing as compared to nonamnion treated group.

These three in vivo studies, utilizing amnion in oral cavity applications, demonstrate some distinct advantages of amnion. First, there was no graft rejection, despite the xenograft nature of the amnion in two of these three studies. Secondly, amnion grafts accelerated healing, while reducing inflammation and acting as a bacterial barrier. Lastly, no interference with bone growth was observed in a model for guided bone regeneration.

*(ii) Clinical Studies*

- Güler et al. in 1997 studied the use of a single layer of lyophilized, gamma irradiated amnion for vestibuloplasty in 20 patients [62]. The graft was sutured in place and no stent was used to cover the graft. Observations of the graft sites 24 hours after amnion application demonstrated a hyperaemic appearance of the mucosal flaps. All patients showed some edema, which resolved by day 7. On day 10, epithelialization of the graft was observed and the amnion graft could not be differentiated. Smooth granulation tissue covered the grafted areas by day 14; and the amnion had completely degraded. At day 21, the grafted areas were completely covered with oral mucosa. In addition, blood flow to the alveolar mucosa was measured in patients by clearance of intramucosal injections of radioactive xenon gas. At day 10, a significant increase in blood flow in the graft was detected, compared with the preoperative state. At 30 days, the blood flow decreased and was not significantly different from normal levels.
- A similar study by Basa et al. in 1987, which used autologous palatal grafts, showed that the blood flow to the grafted area decreased at day 10 and did not return to normal blood flow for 4 weeks postoperatively [63]. At 6 weeks, the blood flow continued to increase and the tissue appeared lighter in color than surrounding mucosa. Güler et al. in 1997 proposed that the angiogenic property of the amnion grafts resulted in more rapid revascularizations and subsequent epithelialization of the grafted areas [62]. Subsequently, healing for the amnion grafts was significantly shorter.
- Samandari et al. in 2004 suggested that the amniotic membrane graft might be used as a potential graft material for vestibuloplasty [64].
- Gurinsky in 2009 reported results of a series of five patients treated with membranes for shallow-to-moderate Miller Classes I and II recession defects [65]. At 12 weeks, an increase in newly generated gingival tissue of 3.2 mm + 1.7 mm was measured. Coverage was 100% in four out of five patients and 88% in the fifth patient.
- Kothiwale et al. in 2009 clinically and radiographically evaluated and compared the efficacy of demineralized freeze-dried bone allograft and bovine derived xenogeneic bone graft with amniotic membrane in the treatment of human periodontal Grade II buccal furcation defects. Results showed significant pocket depth reductions, clinical attachment level gains, and significant improvement in bone fill and percentage gain with both of the materials [66].
- Wallace in 2010 evaluated clinically and histologically the efficacy of a new resorbable, immunoprivileged, self-adhering amniotic membrane for ridge preservation following tooth extraction [67]. Quality of the histologically evident bone formed at 4.5 months was excellent. There was no evidence of resorption of crestal bone height and inflammation, which suggests the potential benefits of using amniotic allograft in guided bone regeneration.
- Sikder et al. in 2010 excised and reconstructed a case of premalignant lesion-leukoplakia of the left buccal mucosa with human amniotic membrane graft [7]. After 4 weeks of grafting procedure, mucosal defect was restored successfully without any complications. Similarly, Ehtaih Sham and Sultana in 2011 used amnion membrane for the reconstruction of a buccal mucosal defect after excision of speckled leukoplakia and found good reconstruction, postoperative function, and aesthetics [68].
- Arai et al. in 2011 showed the clinical usefulness of the hyperdry amniotic membrane as an intraoral wound dressing material [56]. The results suggested that it is biologically acceptable to oral wounds and could be a suitable clinical alternative for the repair of the oral mucosa.
- Rosen in 2011 used a combined approach for correcting both the hard- and soft-tissue deformities around a maxillary canine that included a mineralized bone allograft, recombinant platelet derived growth factor, and a chorion amnion barrier covered by a subepithelial connective tissue graft. The advantages of this particular barrier are that it is extremely thin, measuring 300 mm after full hydration, with the major noncollagenous components being laminins, proteoglycans, and fibronectin, further enhancing its tissue friendly nature [69].
- Kothari et al. in 2012 also concluded that grafts of amniotic membrane are viable and reliable for covering of the raw surface, prevent secondary contraction after vestibuloplasty, and maintain the postoperative vestibular depth [70].
- A clinical trial carried out by Suresh and Gupta in 2013, on a 56-year-old male with vertical recession depth of 2 mm in upper right canine for root coverage and enhancement of gingival biotype by using chorion membrane along with coronally advanced flap, showed 100% root coverage and the soft-tissue biotype enhancement from thin to thick [71].
- Holtzclaw and Toscano in 2013 used amniotic membrane tissue as a barrier membrane for regeneration in the treatment of periodontal intrabony defects in localised moderate to severe chronic periodontitis cases. All patients were treated by thorough degranulation of intrabony periodontal defects and placement of bone allograft covered by amnion-chorion membrane. Clinical measurements 12 months after surgery revealed an average probing depth reduction of 5.06–1.37 mm and clinical attachment level improvement of 4.61–1.29 mm [72]. These membranes being thin in diameter (300 nm) have an advantage over other collagen membranes (700–800 nm) used in guided-tissue regeneration. They adapt better to anatomy of defects and root contours [73].
- H. Singh and H. Singh in 2013 presented a case report on bioactive amniotic membrane as a membrane for the treatment of isolated gingival recession. The results showed significant root coverage with uneventful healing [74].
- Shetty et al. in 2014 compared usage of Platelet-rich Fibrin (PrF) and amniotic membrane in bilaterally occurring multiple Miller Class I recession. 100% root coverage was observed with both of the membranes but the results were stable even after seven months in the amniotic membrane-treated site [75].

## 9. Proposed Mechanism for Gingival Tissue Healing with Amnion Allograft

Amnion has been shown to have exceptional biocompatibility and healing capacity in both the preclinical and clinical studies cited above. The nature of the biocompatibility may be attributed, in part, to the laminin-5 contained in the basement membrane of amnion, as well as to the presence of growth factors and tissue inhibitor of metalloproteinases-1 [17].

## 10. Limitations

1. The use of amniotic membranes requires skill; thus, operator's inexperience is one limitation.
2. There is always an associated risk of infection transmission with transplantation of amniotic membranes. Adequate precautions should be taken and safety criteria should be included in application of these biological membranes [76].
3. Amniotic membranes are fragile membranes, so they need to be dealt with very carefully. Cryopreserved/hyperdry membranes are expensive [76].
4. The procedure associated with use of these membranes is technique-sensitive and also depends on defect morphology. Competent knowledge of wound healing and periodontal regeneration is essential to successfully perform this procedure [72].

## 11. Conclusion

Amniotic membranes have a rich inheritance of collagen types I, IV, V, and VI, proteoglycans, laminin, and fibronectin. Collagen is well tolerated and bioabsorbable, has hemostatic properties, and encourages migration of adjacent autogenous connective tissue and epithelial cells over its surface. Laminins exhibit variety of biological activities including promotion of cell attachment, growth, and differentiation of number of cell types. Fibronectin is involved in many cellular processes including tissue repair, blood clotting, cell migration, and adhesion. Such diverse properties make them a unique novel and potential biomaterial for use in medicine, tissue engineering, stem cell research, repair, and regeneration. Their use in dentistry is also quickly expanding with wide range of applications in Periodontics. Prospective clinical studies are needed to evaluate the efficacy of these membranes in comparison with currently available alternatives. More research and clinical studies are required to completely elucidate their enormous potential. This will help in defining their overall scope and further applications in the field of Periodontics.

## Figures and Tables

**Table 1:** Structure and composition of placental membranes.

Placental membrane	Layers of placental membrane	Extracellular matrix component
Amnion	(1) Epithelium monolayer/amniotic epithelium (2) Basement membrane (3) Compact layer (4) Fibroblast layer (5) Intermediate/spongy layer	Single layer of cells Collagen types III, IV, and V, fibronectin, laminin, and nidogen Collagen types I, III, V, and VI, fibronectin Collagen types I, III, and VI, fibronectin, laminin, and nidogen Collagen types I, III, and IV, proteoglycans

Chorion	(1) Reticular layer (2) Basement membrane (3) Trophoblasts	Collagen types I, III, IV, V, and VI, proteoglycans Collagen type IV, fibronectin, and laminin

**Table 2:** Properties and mechanism of action of amnion membrane.

Immune system	Epithelial tissue	Mesenchymal tissue	Biomechanical properties
Suppression of inflammation [30–39]	Stimulation of growth of epithelial cells [18–27]	TGF-b-suppression of myofibroblastic differentiation [28, 29]	Avascular [17], devoid of cells, no immune response [41–43]

Antibacterial factors and antiviral factors [49–51]	Epidermal growth factor and keratinocyte growth factor [24]	Reduction of scarring [28, 29]	Durable and puncture resistant [56]

Induction of apoptosis of inflammatory cells		Axonal regeneration [77]	Elastic and translucent [68]

		Neural growth factor	Serving as a physical barrier [56]
